# Islet Stellate Cells Isolated from Fibrotic Islet of Goto-Kakizaki Rats Affect Biological Behavior of Beta-Cell

**DOI:** 10.1155/2016/6924593

**Published:** 2015-12-01

**Authors:** Feng-Fei Li, Bi-Jun Chen, Wei Li, Ling Li, Min Zha, S. Zhou, M. G. Bachem, Zi-Lin Sun

**Affiliations:** ^1^Department of Endocrinology, Zhongda Hospital, Institute of Diabetes, School of Medicine, Southeast University, Nanjing 210009, China; ^2^Department of Endocrinology, Nanjing First Hospital, Nanjing Medical University, Nanjing 210012, China; ^3^Department of Clinical Chemistry, University Hospital Ulm, 89081 Ulm, Germany

## Abstract

We previously isolated islet stellate cells (ISCs) from healthy Wistar rat islets. In the present study, we isolated “already primed by diabetic environment” ISCs from islets of Goto-Kakizaki rats, determined the gene profile of these cells, and assessed the effects of these ISCs on beta-cell function and survival. We detected gene expression of ISCs by digital gene expression. INS-1 cell proliferation, apoptosis, and insulin production were measured after being treated with ISCs supernatant (SN). We observed the similar expression pattern of ISCs and PSCs, but 1067 differentially expressed genes. Insulin production in INS-1 cells cultured with ISC-SN was significantly reduced. The 5-ethynyl-2′-deoxyuridine-positive INS-1 cells treated with ISC-SN were decreased. Propidium iodide- (PI-) positive INS-1 cells were 2.6-fold higher than those in control groups. Caspase-3 activity was increased. In conclusion, ISCs presented in fibrotic islet of GK rats might be special PSCs, which impaired beta-cell function and proliferation and increased beta-cell apoptosis.

## 1. Introduction

Type 2 diabetes mellitus (T2DM) has reached pandemic proportions, and current predictions show that this trend will continue [1, 2]. Therefore, achieving a better understanding of this complex disease is imperative. Islet fibrosis in T2DM has received increasing scientific attention [3–10]. Studies showed that pancreatic stellate cells (PSCs) have important functions in islet fibrogenesis in both rodent animal models and human patients with T2DM [6, 7, 11, 12]. In addition, we observed that high glucose aggravates the detrimental effects of pancreatic stellate cells on beta-cell function [13]. To elucidate the underlined mechanisms responsible for islet fibrosis in the late stage of T2DM, we find that endocrine pancreatic islets contain cells resembling PSCs and suggest these may contribute to islet fibrosis in T2DM [14].

In the normal pancreas, PSCs are quiescent and are found in low abundance [15, 16]. Upon pancreatic injury or pancreatic inflammation, PSCs lose their vitamin A stores and transform from “activated” into myofibroblast-like phenotypes, which highly proliferate, migrate, synthesize, and secrete excessive amounts of the extracellular matrix (ECM) proteins, resulting in tissue fibrosis [15–23].

The function of PSCs in islet fibrosis has been the subject of several studies for years.* In vivo* studies have shown that PSCs are present in rat islets and are involved in islet fibrogenesis in several animal models of T2DM [10, 11, 24–27]. In humans, PSCs are also present in islets of T2DM patients and possibly have a function in the progression of islet fibrosis [11]. Fibrosis is one of the major factors leading to progressive pancreatic beta-cell loss and dysfunction [6, 11, 28–33]. Efforts have been made for developing antifibrotic strategies to ameliorate islet fibrosis and the progression of T2DM [6, 12, 34–36].* In vivo* studies showed that the attenuation of PSC activation reduces islet fibrosis [6, 12, 26, 36] or increases insulin content [26].

Kikuta et al. recently reported that indirect coculture of RIN-5F cells with PSCs results in decreased insulin production and increased cell apoptosis [37], and very recently published data showed that activated PSCs can impair pancreatic islet function in mice [38]. We observed that PSCs transplantation exacerbated the impaired *β*-cell function in GK rats [13] and demonstrated that PSCs resemble cells within endocrine pancreatic islets [14]. However, in T2DM, the PSCs that presented intra-/peri-islets were exposed to the islet niche, such as marked hyperglycemia [28, 39, 40], oxidative stress [41–43], and inflammation [4, 28, 39]. These factors could affect PSCs activation and proliferation and subsequently stimulate the production of endogenous inflammatory mediators in PSCs [17]. Thus, PSCs were already “activated” by the environmental conditions of T2DM* in vivo*. Therefore, we isolated stellate cell from fibrotic islets of Goto-Kakizaki (GK) rats, and our previous data pushed us to question whether these population cells were a special type of PSCs and which effect is on biological behavior of beta-cells.

## 2. Methods

### 2.1. Animals and Ethics Statement

Rats were housed in cages (three rats per cage) under a 12 h/12 h light/dark cycle. Rats were given free access to food and water ad libitum. Animal experiments were approved by the Southeast University Animal Care and Use Committee according to institutional guidelines and national animal welfare.

### 2.2. Isolation of PSCs

PSCs were isolated from 8-week-old Wistar rats as described previously [16]. PSCs were cultured in Dulbecco's modified Eagle's medium (DMEM)/Ham's F12 (1 : 1 v/v) containing 10% fetal bovine serum (FBS) (Invitrogen, Carlsbad, USA). Cell purity was assessed by immunostaining for vimentin (100%), *α*-SMA (>95%), and desmin (20–50%).

### 2.3. Isolation and Culture of ISCs and Preparation of ISC-SN

Islets were isolated from four-month-old male GK rat pancreas as previously described [4]. In brief, pancreas tissues were digested with collagenase V (1 mg/mL, w/v) (Sigma, St. Louis, MO, USA) at 37°C for 15 min to 18 min. Islets were purified by handpicking twice under a stereomicroscope. Then, islets were precultured in RPMI-1640 supplemented with L-glutamine containing 10% (v/v) fetal bovine serum (FBS) (Invitrogen, Carlsbad, CA, USA) overnight followed by handpicking.

After 48 h in culture, ISCs began to grow out of GK islets. After 5 d, cells were subcultured in DMEM/Ham's F12 (1 : 1, v/v) containing 10% FBS. Cells at passages 3 to 8 were used for experiments.

To prepare ISC-SN, cells grown near confluence were cultured with DMEM/F12 serum-free medium plus 0.2% BSA for another 48 h. The culture medium was collected, centrifuged, filtered, and stored at −80°C until use.

### 2.4. Immunofluorescence Staining

After shortly being washed with cold phosphate buffer saline (PBS), cells were fixed with 4% paraformaldehyde at room temperature for 20 min and subsequently stained with primary antibodies at the following dilutions: *α*-SMA, (1 : 100, DAKO, Hamburg, Germany), vimentin (1 : 200, DAKO), and desmin (1 : 50, DAKO), followed by fluorescent secondary antibodies (DAKO). The nuclei were then counterstained with bisbenzimide. Photos of eight different areas in each well were taken using a microscope with 100x magnification. A minimum of 500 cells in each experimental group was analyzed (*n* = 3).

### 2.5. Digital Gene Expression (DGE) Profile of ISCs and PSCs

The passage 3 ISCs and PSCs were used for DGE analysis. DGE was performed by the BGI Tech (Shenzhen, China).

### 2.6. Treatment of INS-1 Cells with ISC-SN

Insulin-producing *β*-cell line (INS-1 832/13) cells were seeded into RPMI-1640 medium with L-glutamine containing 10% FBS. Upon reaching confluence, INS-1 cells were treated with or without 35% ISC-SN for up to 48 h. After exposure to ISC-SN, INS-1 cells were washed in RPMI 1640, and the following experiments were performed, representing *n* = 3 to 4.

#### 2.6.1. 5-Ethynyl-2′-deoxyuridine (EdU) Incorporation Assay

EdU incorporation assay was performed as previously described [44]. In brief, EdU (Molecular Probes, Eugene, OR, USA) was added 4 h before the experiments ended and stained. Photos of eight different areas in each well were taken using a microscope with 200x magnification. The average percentage of EdU^+^/DAPI^+^ was calculated.

#### 2.6.2. Propidium Iodide (PI) Staining

After removing the medium, INS-1 cells were incubated with PI (20 *μ*g/mL) and Hoechst (2 *μ*g/mL) (Sigma, St. Louis, MO, USA) for 10 min in the dark. Photos of eight different areas in each well were taken using a microscope with 200x magnification. The average PI staining rate was expressed as the percentage of PI-positive nuclei compared with the total cell count.

#### 2.6.3. Caspase-3 Fluorometric Assay (CFA)

CFA was performed according to the manufacturer's instructions (R&D, Minneapolis, MN, USA). CFA was performed in a 96-well flat-bottom microplate. After incubation with the substrate for 1.5 h, the plate was read on a fluorescent microplate reader equipped with filters set at 400 nm/505 nm (excitation/emission wavelengths). Data were normalized with total protein content in each well and expressed as OD/*μ*g/*μ*L.

#### 2.6.4. Quantitative RT-PCR (qRT-PCR)

Total RNA of INS-1 cells was extracted using TRIzol reagent (Invitrogen). The primer sequences used for amplification of genes encoding insulin and GAPDH are listed in Table 1. qRT-PCR analyses were performed using a standard SYBR-Green PCR kit protocol on a Step One Plus system (Applied Biosystems, Foster City, CA, USA) according to the manufacturer's instructions. The relative level of insulin transcripts was calculated and normalized to GAPDH, with at least four repeats per experimental group.

#### 2.6.5. Potassium-Stimulated Insulin Secretion (KSIS) Assay

Measurements of potassium-stimulated insulin secretion (KSIS) by INS-1 cells were performed as described [13, 45]. Briefly, After preincubation in glucose-free Krebs-Ringer bicarbonate buffer for 1 h, cells were treated with low glucose (2.8 mmol/L) and high potassium (50 mmol/L) for 1 h. Insulin secretion after stimulation and insulin content in the cell lysate were measured using an insulin radioimmunoassay kit (Beijing Technology Company, Beijing, China). Data were normalized to the cellular insulin content and expressed as a percentage.

#### 2.6.6. Measurement of Cytokines in ISC-SN

To determine the cytokines present in ISC-SN, the RayBio Biotin Label-Based Rat Antibody Array 1 (RayBiotech, Norcross, GA, USA) was performed according to the manufacturer's recommendations.

### 2.7. Statistical Analysis

Data are presented as the mean ± SE. Statistical significance was determined by unpaired Student's *t*-test or ANOVA, followed by the Bonferroni–Dunn post hoc test. *P* < 0.05 was considered statistically significant. All statistical analyses were performed using the Statistical Product and Services Solutions (SPSS) package (Version 11.5, SPSS Science, Chicago, IL, USA).

## 3. Results

### 3.1. Isolation and Characterization of ISCs

**Figure 1:**
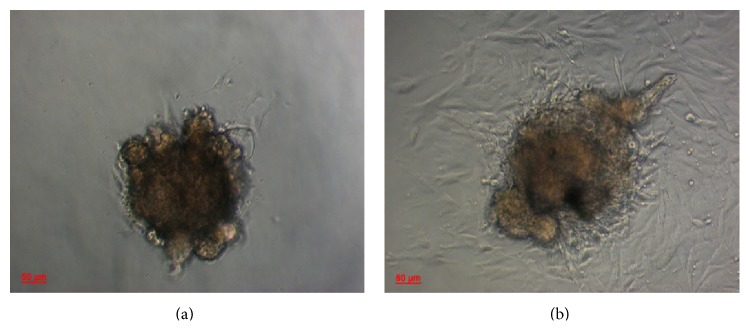
(a) Some stellate shape-like cells began to grow out of four-month-old GK rat islets after 48 h of culture. (b) These cells exponentially proliferated and migrated out from the islets after 72 h, at 100x magnification (*n* = 3).

**Figure 2:**
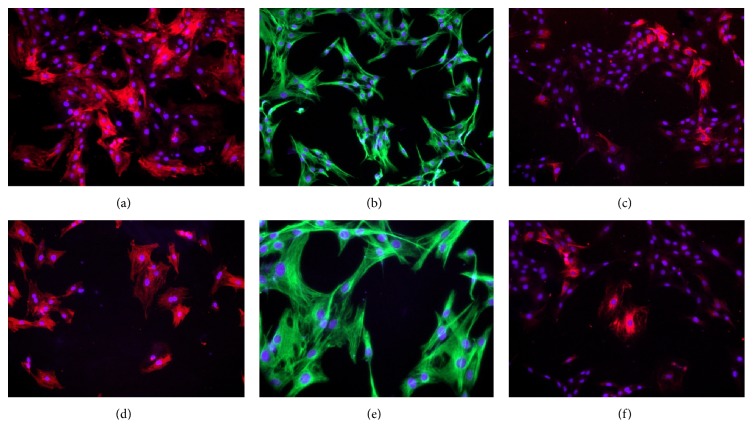
Stellate shape-like cell expression of (a) *α*-SMA, (b) vimentin, and (c) desmin and PSC expression of (d) *α*-SMA, (e) vimentin, and (f) desmin. Representative images for *α*-SMA and desmin staining were red, and those for vimentin were green at 100x magnification.

After 48 h of culture, cells with triangular shapes and large nuclei began to grow out of GK rat islets (Figure 1(a)). When the culture period was extended, these cells migrated away from the islet (Figure 1(b)). In addition, these stellate cells had a shorter doubling time (28 h). To determine these cells markers, immunofluorescence staining was performed. The ISC was positive for *α*-SMA (Figure 2(a)), vimentin (Figure 2(b)), and desmin (Figure 2(c)), thereby resembling the pattern of PSCs (Figures 2(d), 2(e), and 2(f)).

### 3.2. The DGE Profile of ISC Compared with PSC

Taking advantage of the DGE performed by the BGI Tech, we observed that the relative mRNA expression levels of ISCs were similar to PSCs mRNA expression pattern in intermediate filaments (*α-SMA*,* vimentin*, and* desmin*), ECM (*Procollagen, Pro-α-1 collagen type, Collagen α-1 type IX, Collagen α-1 type XI, Collagen α-1 type III, Fibronectin, Laminin, Tenascin, TIMP1, TIMP2, MMP1, MMP2, MMP14, RECK*, and* TUBA6*), cytokines (*IL-1β, IL-6, IL-7, IL-15, IL-18, TGF-α, TGF-β1, TGF-β2, TGF-β binding protein 2, TGF-β binding protein 3, HGF, PDGF-A, PDGF-B, CTGF, RANTES, MCP-1, ET-1*, and* VEGF*), signal transduction (*Smad1, Smad2, Smad7, Smad8, ERK1, ERK3*, and* ERK5*), integrins (*integrin α-1, integrin α-E2, integrin α-M, integrin α-v, integrin β, integrin β-3, integrin β-5*, and* integrin associated protein*), cytokine receptors (*TGFβR type1, PDGFRβ, PGFR1β, FGFR1, IL-3Rβ*, and* ActivinR*), and* PPARγ*. However, there were 1657 genes differentially expressed (600 genes upregulated and 1057 genes downregulated) of ISCs compared with that of PSCs, which enriched GO terms in metabolic pathways (196, 13.75%), pathways in cancer (63, 4.42%), MAPK signaling pathway (48, 3.37%), focal adhesion (46, 3.23%), regulation of actin cytoskeleton (44, 3.09%), biosynthesis of secondary metabolites (39, 2.74%), Alzheimer's disease (38, 2.67%), cell cycle (29, 2.04%), tight junction (37, 2.6%), vascular smooth muscle contraction (34, 2.39%), microbial metabolism in diverse environments (34, 2.39%), and chemokine signaling pathway (33, 2.32%), as well as Wnt signaling pathway (32, 2.5%) (for more information please see Supplementary Table 1 in the Supplementary Material available online at http://dx.doi.org/10.1155/2016/6924593).

### 3.3. ISC-SN Impaired INS-1 Cell Survival

**Figure 3:**
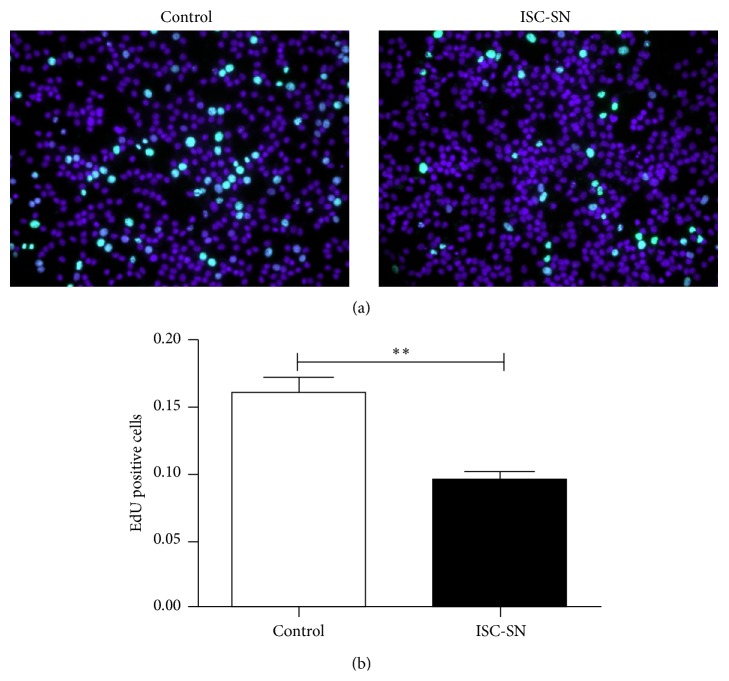
Effect of ISC-SN on INS-1 cell proliferation. (a) Cell proliferation was performed using EdU incorporation assay after treatment of INS-1 cells with ISC conditional medium for 48 h. Representative images for EdU staining (green) and nuclei labeled by DAPI (blue) at 200x magnification. (b) Data were expressed as mean ± SE (*n* = 3), ^*∗∗*^ *P* < 0.01.

Using EdU incorporation assay, the effects of ISC-SN on INS-1 cell proliferation were assessed. The proliferative capacity of INS-1 cells treated with ISC-SN (0.09 ± 0.01) was more significantly reduced than that in the control group (0.16 ± 0.02, *P* < 0.01) (Figures 3(a) and 3(b)).

**Figure 4:**
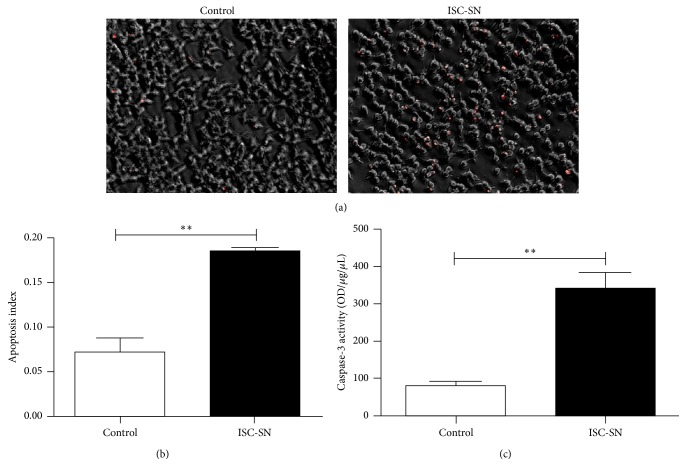
Effect of ISC-SN on INS-1 cell apoptosis. (a) Cell apoptosis was determined by PI staining (red), (b) PI-staining index, and (c) caspase-3 activity in INS-1 cells cultured with ISC-SN for 48 h. Data are expressed as mean ± SE (*n* = 3), ^*∗∗*^ *P* < 0.01.

To observe the effects of ISC-SN on INS-1 cell survival, apoptosis in INS-1 cells incubated with ISC-SN was detected by PI staining and CFA. As shown in Figure 4(a), a higher number of PI-positive INS-1 cells were observed in ISC-SN group. Quantitative analysis of PI-positive index (Figure 4(b)) showed that the dead cells in the ISC-SN group (0.18 ± 0.03) were 2.6-fold higher than those in the control (0.07 ± 0.02) groups. Caspase-3 activity in the ISC-SN groups (338.09 ± 62.51) was significantly higher than that in control groups (80.40 ± 16.59, *P* < 0.01) (Figure 4(c)).

### 3.4. ISC-SN Reduced Insulin Secretion of INS-1 Cells

**Figure 5:**
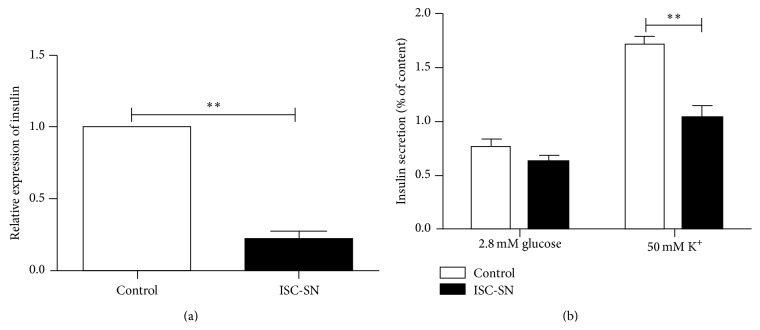
Effects of ISC-SN on insulin mRNA transcript and INS-1 cell function. (a) qRT-PCR analysis of insulin mRNA expression and (b) KSIS assay on INS-1 cells cultured with ISC-SN for 24 h. Insulin mRNA data were normalized by GAPDH gene, and insulin secretion data were normalized by cellular insulin content. Data are expressed as mean ± SE (*n* = 4 to 5), ^*∗∗*^ *P* < 0.01.

To investigate the effects of ISC-SN on beta-cell function, insulin mRNA expression and secretion in INS-1 cells incubated with ISC-SN for 24 h were evaluated.  Figure 5(a) showed that the insulin mRNA expression in INS-1 cells treated with ISC-SN (0.21 ± 0.10) was more significantly decreased than that in the control group (*P* < 0.01). In response to potassium challenge, insulin secretion in INS-1 cells incubated with ISC-SN (1.03% ± 0.23%) was more significantly reduced than that in control groups (1.71% ± 0.17% *μ*IU/mL, *P* < 0.01) (Figure 5(b)).

### 3.5. Cytokine Profile of ISC-SN

To determine the cytokines that were highly expressed in ISCs-SN, RayBio Biotin Label-Based Rat Antibody Array 1 was performed. Cytokines, such as integrin *α*M*β*2, IFN-*γ*, MCP-1, MMP-2, PDGF, TNF-*α*, TLR4, and TROY, were highly expressed in both ISCs-SN and PSCs-SN (Table 2), whereas CD106, IL-1*β*, CXCR4, IL-3, IL-5, and MIF were highly expressed in ISCs-SN (Table 3).

## 4. Discussion

In this study, we suggested an effective method for isolating ISCs from fibrotic islets of four-month-old GK rats. We observed a significant overlap and differentially expressed genes of ISCs and PSCs. In addition, ISCs exerted deleterious effects on beta-cells.

Previous studies have proven the existence of islet fibrosis in T2DM patients and rodent animal models, including GK rats [3, 4, 8], a spontaneous T2DM model [46, 47].* In vivo* studies showed that PSCs are present in rat islets and are critically important in the progression of islet fibrogenesis [10, 11, 24–27]. Evidence for the involvement of PSCs in islet fibrosis is mainly based on staining of *α*-SMA and/or GFAP for marker detection [25, 48, 49]. Islet fibrosis is possibly caused by other cell types that express the same markers, such as circulating fibrocytes. To determine which cell types are present in fibrotic islets of T2DM, islets from four-month-old GK rats were isolated and cultured. Although most islets in 16-week-old GK rats were significantly deformed with massive fibrosis, 90 ± 16 islets were harvested per pancreas using handpicking after mild digestion with collagenase V for 15 min to 18 min. As shown in Figure 1, stellate-like cells began to grow out of the islets after 48 h of culture, and these cells often exhibited a triangular shape and a large nucleus. Immunofluorescence staining demonstrated that these cells were positive for *α*-SMA, vimentin, and desmin, thereby resembling the protein expression profile of PSCs [15, 16]. Given that these cells were isolated from fibrotic islets of GK rats, which were presumably already influenced by the surrounding islet microenvironment in T2DM, were highly similar to activated PSCs. The isolation of ISCs from fibrotic islets in GK rats may provide a tool to investigate the interactions between ISCs and pancreatic beta-cell* in vitro*.

The interesting results in this study are the deleterious effects of ISC on INS-1 cell function and survival. Previous study has clearly shown that PSCs reduced insulin expression and induced apoptosis in pancreatic beta-cell [37], and high glucose aggravates the detrimental effects of PSCs on beta-cell function [13]. In this study, our data indicated that ISC also exhibited deleterious effects on INS-1 beta-cells by reducing insulin mRNA transcription and inhibiting insulin secretion. In addition, ISCs significantly decreased cell proliferation and increased cell apoptosis. To determine the factors that possibly contribute to the effect of ISCs on INS-1 cells, cytokine profiles in ISCs were assessed. ISCs-SN was shown to contain high levels of cytokines, such as IFN-*γ* and TNF-*α*, which are well-known factors mediating or promoting cell death [50–55]. Our data also show that ISCs-SN contained higher levels of cytokines than PSCs-SN, such as IL-1*β*, which can impair beta-cell function and induce cell death at higher concentrations [56].

## 5. Conclusion

In conclusion, our* in vitro* study shows that fibrotic islets of GK rats contain a population of stellate cells resembling but not identical to PSCs, which exerted deleterious effects on beta-cells by inducing beta-cell death and suppressing insulin production and cell proliferation.

## Supplementary Material

Supplementary Table 1: DGE was performed to detect ISC and PSC cDNA expression. ISCs up and down-regulated 1657 genes compared with PSCs (≥ 2.0-fold of or ＜-2.0-fold of was considered as differentially expressed).

## Figures and Tables

**Table 1:** Primer sequences of genes encoding insulin and GAPDH.

Gene	Primers
Insulin	5′-ATTGTTCCAACATGGCCCTGT-3′
	5′-TTGCAGTAGTTCTCCAGTT-3′

GAPDH	5′-TGTTCCTACCCCCAATGTGTCCGTC-3′
	5′-CTGGTCCTCAGTGTAGCCCAAGATG-3′

**Table 2:** Cytokines were highly expressed in both ISC-SN and PSC-SN. ISCs-SN/PSC-SN was quantified by mean fluorescence intensity (MFI) using RayBio Biotin Label-Based Rat Antibody Array 1 assay. MFI ≥ 1.5-fold of negative control was considered as highly expressed.

	ISCs-SN	PSCs-SN
	(MFI)	(MFI)
Negative control	5828.58	6319.81
Integrin alpha M beta 2	88599.0	75866.5
IFN-gamma	17506.0	16974.0
MCP-1	17223.0	24933.5
MMP-2	13303.0	22384.5
PDGF	20689.0	28034.5
TNF-alpha	17654.5	24232.0
TLR4	69218.5	48604.0
TROY	96330.0	104265.0

**Table 3:** Cytokines were highly expressed in ISCs-SN than in PSCs-SN. MFI of ISCs-SN ≥ 1.5-fold of PSCs-SN was considered as highly expressed.

	ISCs-SN	PSCs-SN	Fold
	(MFI)	(MFI)	
Negative control	5828.5	6319.8	
CD106	47241.0	10725.0	4.40
IL-1*β*	22605.5	10431.0	2.24
CXCR4	23640.0	10119.5	2.34
ICAM-1/CD54	41550.0	12306.5	3.38
ICK	23586.0	11006.5	2.14
IL-3	12685.0	5296.0	2.40
IL-5	10394.0	6536.5	1.59
MIF	37598.0	17121.5	2.20

## References

[1] Danaei G., Finucane M. M., Lu Y. (2011). National, regional, and global trends in fasting plasma glucose and diabetes prevalence since 1980: systematic analysis of health examination surveys and epidemiological studies with 370 country-years and 2·7 million participants. *The Lancet*.

[2] Ning G., Hong J., Bi Y. (2009). Progress in diabetes research in China. *Journal of Diabetes*.

[3] Guenifi A., Abdel-Halim S. M., Hoog A., Falkmer S., Ostenson C.-G. (1995). Preserved *β*-cell density in the endocrine pancreas of young, spontaneously diabetic Goto-Kakizaki (GK) rats. *Pancreas*.

[4] Homo-Delarche F., Calderari S., Irminger J. C. (2006). Islet inflammation and fibrosis in a spontaneous model of type 2 diabetes, the GK rat. *Diabetes*.

[5] Kawano K., Hirashima T., Mori S., Saitoh Y., Kurosumi M., Natori T. (1992). Spontaneous long-term hyperglycemic rat with diabetic complications: Otsuka Long-Evans Tokushima Fatty (OLETF) strain. *Diabetes*.

[6] Ko S.-H., Kwon H.-S., Kim S.-R. (2004). Ramipril treatment suppresses islet fibrosis in Otsuka Long-Evans Tokushima fatty rats. *Biochemical and Biophysical Research Communications*.

[7] Masuyama T., Komeda K., Hara A. (2004). Chronological characterization of diabetes development in male Spontaneously Diabetic Torii rats. *Biochemical and Biophysical Research Communications*.

[8] Movassat J., Saulnier C., Serradas P., Portha B. (1997). Impaired development of pancreatic beta-cell mass is a primary event during the progression to diabetes in the GK rat. *Diabetologia*.

[9] Pick A., Clark J., Kubstrup C. (1998). Role of apoptosis in failure of beta-cell mass compensation for insulin resistance and beta-cell defects in the male Zucker diabetic fatty rat. *Diabetes*.

[10] Yoshikawa H., Kihara Y., Taguchi M., Yamaguchi T., Nakamura H., Otsuki M. (2002). Role of TGF-*β*1 in the development of pancreatic fibrosis in Otsuka Long-Evans Tokushima Fatty rats. *American Journal of Physiology—Gastrointestinal and Liver Physiology*.

[11] Kim J.-W., Ko S.-H., Cho J.-H. (2008). Loss of beta-cells with fibrotic islet destruction in type 2 diabetes mellitus. *Frontiers in Bioscience*.

[12] Tikellis C., Wookey P. J., Candido R., Andrikopoulos S., Thomas M. C., Cooper M. E. (2004). Improved islet morphology after blockade of the renin-angiotensin system in the ZDF rat. *Diabetes*.

[13] Zha M., Xu W., Zhai Q., Li F., Chen B., Sun Z. (2014). High glucose aggravates the detrimental effects of pancreatic stellate cells on beta-cell function. *International Journal of Endocrinology*.

[14] Zha M., Li F., Xu W., Chen B., Sun Z. (2014). Isolation and characterization of islet stellate cells in rat. *Islets*.

[15] Apte M. V., Haber P. S., Applegate T. L. (1998). Periacinar stellate shaped cells in rat pancreas: identification, isolation, and culture. *Gut*.

[16] Bachem M. G., Schneider E., Gross H. (1998). Identification, culture, and characterization of pancreatic stellate cells in rats and humans. *Gastroenterology*.

[17] Apte M., Pirola R., Wilson J. (2011). The fibrosis of chronic pancreatitis: new insights into the role of pancreatic stellate cells. *Antioxidants and Redox Signaling*.

[18] Apte M. V., Park S., Phillips P. A. (2004). Desmoplastic reaction in pancreatic cancer: role of pancreatic stellate cells. *Pancreas*.

[19] Apte M. V., Wilson J. S. (2003). Alcohol-induced pancreatic injury. *Bailliere's Best Practice and Research in Clinical Gastroenterology*.

[20] Apte M. V., Wilson J. S. (2012). Dangerous liaisons: pancreatic stellate cells and pancreatic cancer cells. *Journal of Gastroenterology and Hepatology*.

[21] Bachem M. G., Zhou Z., Zhou S., Siech M. (2006). Role of stellate cells in pancreatic fibrogenesis associated with acute and chronic pancreatitis. *Journal of Gastroenterology and Hepatology*.

[22] Haber P. S., Keogh G. W., Apte M. V. (1999). Activation of pancreatic stellate cells in human and experimental pancreatic fibrosis. *American Journal of Pathology*.

[23] McCarroll J. A., Phillips P. A., Santucci N., Pirola R. C., Wilson J. S., Apte M. V. (2006). Vitamin A inhibits pancreatic stellate cell activation: implications for treatment of pancreatic fibrosis. *Gut*.

[24] Klonowski-Stumpe H., Fischer R., Reinehr R., Lüthen R., Häussinger D. (2002). Apoptosis in activated rat pancreatic stellate cells. *The American Journal of Physiology—Gastrointestinal and Liver Physiology*.

[25] Koyama M., Wada R., Mizukami H. (2000). Inhibition of progressive reduction of islet beta-cell mass in spontaneously diabetic Goto-Kakizaki rats by alpha-glucosidase inhibitor. *Metabolism: Clinical and Experimental*.

[26] Saito R., Yamada S., Yamamoto Y. (2012). Conophylline suppresses pancreatic stellate cells and improves islet fibrosis in Goto-Kakizaki rats. *Endocrinology*.

[27] Yokota T., Denham W., Murayama K., Pelham C., Joehl R., Bell R. H. (2002). Pancreatic stellate cell activation and MMP production in experimental pancreatic fibrosis. *Journal of Surgical Research*.

[28] Ehses J. A., Lacraz G., Giroix M.-H. (2009). IL-1 antagonism reduces hyperglycemia and tissue inflammation in the type 2 diabetic GK rat. *Proceedings of the National Academy of Sciences of the United States of America*.

[29] Hayden M. R., Sowers J. R. (2007). Isletopathy in type 2 diabetes mellitus: implications of islet RAS, islet fibrosis, islet amyloid, remodeling, and oxidative stress. *Antioxidants & Redox Signaling*.

[30] Hong O.-K., Lee S.-H., Rhee M. (2007). Hyperglycemia and hyperinsulinemia have additive effects on activation and proliferation of pancreatic stellate cells: Possible explanation of islet-specific fibrosis in type 2 diabetes mellitus. *Journal of Cellular Biochemistry*.

[31] Johnson J. D., Luciani D. S. (2010). Mechanisms of pancreatic beta-cell apoptosis in diabetes and its therapies. *Advances in Experimental Medicine and Biology*.

[32] Kaihara M., Nakamura Y., Sugimoto T. (2009). Olmesartan and temocapril prevented the development of hyperglycemia and the deterioration of pancreatic islet morphology in Otsuka-Long-Evans-Tokushima Fatty rats. *Acta Medica Okayama*.

[33] Lee E., Ryu G. R., Ko S.-H. (2011). Antioxidant treatment may protect pancreatic beta cells through the attenuation of islet fibrosis in an animal model of type 2 diabetes. *Biochemical and Biophysical Research Communications*.

[34] Cooper M. E., Tikellis C., Thomas M. C. (2006). Preventing diabetes in patients with hypertension: one more reason to block the renin-angiotensin system. *Journal of Hypertension*.

[35] Li F., Chen B., Li L. (2014). INS-1 cells inhibit the production of extracellular matrix from pancreatic stellate cells. *Journal of Molecular Histology*.

[36] Mizukami H., Wada R., Yonezawa A., Sugawara A., Yagihashi S. (2008). Suppression of post-prandial hyperglycaemia by pioglitazone improved islet fibrosis and macrophage migration in the Goto-Kakizaki rat. *Diabetes, Obesity and Metabolism*.

[37] Kikuta K., Masamune A., Hamada S., Takikawa T., Nakano E., Shimosegawa T. (2013). Pancreatic stellate cells reduce insulin expression and induce apoptosis in pancreatic beta-cells. *Biochemical and Biophysical Research Communications*.

[38] Zang G., Sandberg M., Carlsson P. O., Welsh N., Jansson L., Barbu A. (2015). Activated pancreatic stellate cells can impair pancreatic islet function in mice. *Upsala Journal of Medical Sciences*.

[39] Portha B., Lacraz G., Chavey A. (2010). Islet structure and function in the GK rat. *Advances in Experimental Medicine and Biology*.

[40] Prentki M., Nolan C. J. (2006). Islet *β* cell failure in type 2 diabetes. *The Journal of Clinical Investigation*.

[41] Koyama M., Wada R.-I., Sakuraba H., Mizukami H., Yagihashi S. (1998). Accelerated loss of islet *β* cells in sucrose-fed Goto-Kakizaki rats, a genetic model of non-insulin-dependent diabetes mellitus. *The American Journal of Pathology*.

[42] Lacraz G., Giroix M.-H., Kassis N. (2009). Islet endothelial activation and oxidative stress gene expression is reduced by IL-1Ra treatment in the type 2 diabetic GK rat. *PLoS ONE*.

[43] Mosén H., Östenson C.-G., Lundquist I. (2008). Impaired glucose-stimulated insulin secretion in the GK rat is associated with abnormalities in islet nitric oxide production. *Regulatory Peptides*.

[44] Yu Y., Arora A., Min W., Roifman C. M., Grunebaum E. (2009). EdU incorporation is an alternative non-radioactive assay to [^3^H]thymidine uptake for in vitro measurement of mice T-cell proliferations. *Journal of Immunological Methods*.

[45] Kato T., Shimano H., Yamamoto T. (2006). Granuphilin is activated by SREBP-1c and involved in impaired insulin secretion in diabetic mice. *Cell Metabolism*.

[46] Portha B. (2005). Programmed disorders of *β*-cell development and function as one cause for type 2 diabetes? The GK rat paradigm. *Diabetes/Metabolism Research and Reviews*.

[47] Portha B., Giroix M.-H., Serradas P. (2001). Beta-cell function and viability in the spontaneously diabetic GK rat: information from the GK/Par colony. *Diabetes*.

[48] Rajkumar V. S., Howell K., Csiszar K., Denton C. P., Black C. M., Abraham D. J. (2005). Shared expression of phenotypic markers in systemic sclerosis indicates a convergence of pericytes and fibroblasts to a myofibroblast lineage in fibrosis. *Arthritis Research & Therapy*.

[49] Skalli O., Pelte M.-F., Peclet M.-C. (1989). *α*-smooth muscle actin, a differentiation marker of smooth muscle cells, is present in microfilamentous bundles of pericytes. *Journal of Histochemistry and Cytochemistry*.

[50] Karlsen A. E., Rønn S. G., Lindberg K. (2001). Suppressor of cytokine signaling 3 (SOCS-3) protects *β*-cells against interleukin-1*β*- and interferon-*γ*-mediated toxicity. *Proceedings of the National Academy of Sciences of the United States of America*.

[51] Krammer P. H. (2000). CD95's deadly mission in the immune system. *Nature*.

[52] Mandrup-Poulsen T. (2001). *β*-cell apoptosis: stimuli and signaling. *Diabetes*.

[53] Ou D., Metzger D. L., Wang X., Huang J., Pozzilli P., Tingle A. J. (2002). TNF-related apoptosis-inducing ligand death pathway-mediated human beta-cell destruction. *Diabetologia*.

[54] Stephens L. A., Thomas H. E., Ming L., Darwiche M. G. R., Volodin L., Kay T. W. H. (1999). Tumor necrosis factor-*α*-activated cell death pathways in NIT-1 insulinoma cells and primary pancreatic *β* cells. *Endocrinology*.

[55] Wallach D., Boldin M., Goncharov T. (1996). Exploring cell death mechanisms by analyzing signaling cascades of the TNF/NGF receptor family. *Behring Institute Mitteilungen*.

[56] Maedler K., Schumann D. M., Sauter N. (2006). Low concentration of interleukin-1beta induces FLICE-inhibitory protein-mediated beta-cell proliferation in human pancreatic islets. *Diabetes*.

