# Supplementary material for: Islet Stellate Cells Isolated from Fibrotic Islet of Goto-Kakizaki Rats Affect Biological Behavior of Beta-Cell
Source: J Diabetes Res. 2015 Dec 1;2016:6924593. doi: 10.1155/2016/6924593 (PMC4678093; doi:10.1155/2016/6924593)
Supplement: Supplementary file 1 — Supplementary Table 1: DGE was performed to detect ISC and PSC cDNA expression. ISCs up and down-regulated 1657 genes compared with PSCs (≥ 2.0-fold of or ＜-2.0-fold of was considered as differentially expressed). [file 6924593.f1.pdf]

Supplementary table 1. Differential expression genes between ISCs and PSCs

ISCs (P3) *vs.* PSCs (P3)		ISCs (P3) *vs.* PSCs (P3)		ISCs (P3) *vs.* PSCs (P3)	
Symbol	Fold of	Symbol	Fold of	Symbol	Fold of
Serpina12	12.81278	*Asb2*	-10.9084	*Reps1*	-3.09692
Cd180	12.55603	*Shox2*	-10.4105	*Fxr1*	-3.05802
Lhx8	11.58026	*Bhlhe22*	-10.2981	*Gpm6b*	-3.03123
Fam180a	10.39232	*Tnfrsf21*	-10.218	*Olfm1*	-3.02828
Igfbp2	10.37395	*Clec14a*	-10.218	*Ddah2*	-3.01969
Abhd16a	10.25857	*RGD1306576*	-10.1111	*Kif5c*	-3.00088
Atp2a1	10.21796	*Ifitm1*	-10.0888	*Thy1*	-2.99609
Cmklr1	9.296916	*Fxyd6*	-9.97154	*Serinc3*	-2.99563
Mgll	9.257388	*Nefm*	-9.94691	*Cyp4b1*	-2.97143
Cfb	9.257388	*Dcdc2*	-9.94691	*Uap1*	-2.96851
Smoc1	9.216746	*Ccl12*	-9.94691	*Itm2a*	-2.96347
Fam111a	9.131857	*Hoxb5*	-9.92184	*Sap18*	-2.94067
Il16	9.087463	*Arhgdib*	-9.92184	*Nox1*	-2.9386
Ereg	8.945444	*LOC24906*	-9.89633	*Crebzf*	-2.9386
Dhrsx	8.945444	*Sectm1b*	-9.84392	*Rala*	-2.93695
Orm1	8.894818	*Mcam*	-9.81698	*Grem2*	-2.93341
Fcrla	8.84235	*Tmem178*	-9.73302	*Ccdc82*	-2.926
Ano1	8.787903	*Ggt5*	-9.64386	*Cacna1c*	-2.926
Cdc6	8.787903	*Cd40*	-9.54689	*Nrn1*	-2.9176
Tbx1	8.731319	*Rtn1*	-9.51373	*Cyld*	-2.88753
Tnn	8.672425	*Atp8b1*	-9.44501	*Id2*	-2.88753
Bst2	8.611025	*Dchs1*	-9.40939	*Procr*	-2.85065
Lbp	8.601734	*Tmem132e*	-9.40939	*Lrrn4cl*	-2.848
Mrpl43	8.546894	*RGD1562533*	-9.40939	*Ppp1r14a*	-2.84228
Tiam1	8.546894	*Stac2*	-9.33539	*Mfap5*	-2.82818
Lrp2	8.47978	*Kras*	-9.33539	*Tspan7*	-2.82216
Cenpf	8.409391	*Serpinb6b*	-9.33539	*Wfdc1*	-2.81734
Oscp1	8.409391	*Nap1l2*	-9.29692	*Lmcd1*	-2.81654
Dagla	8.33539	*Slc15a3*	-9.21675	*Dapk2*	-2.80735
Icam4	8.257388	*Ptn*	-9.21675	*C1galt1*	-2.80735
Pold1	8.257388	*Egflam*	-9.21675	*Adm2*	-2.80735
Adhfe1	8.174926	*Aard*	-9.1783	*Ntm*	-2.76637
Vcam1	8.051363	*Cd24*	-9.17493	*Peli1*	-2.75646
Nostrin	7.994353	*Has2*	-9.13186	*Ccdc127*	-2.75489
Iqgap3	7.994353	*Nog*	-9.08746	*Miip*	-2.75489
Aass	7.894818	*Akap5*	-9.04166	*Echdc2*	-2.75489
Repin1	7.894818	*RGD1304810*	-8.94544	*Rsrc1*	-2.74577
Aldh3a1	7.894818	*Ptafr*	-8.89482	*Acyp2*	-2.74543
Dmrta1	7.894818	*Ankrd13d*	-8.89482	*Anxa3*	-2.74428
Prss12	7.894818	*Mat1a*	-8.89482	*Cnih2*	-2.74348

Mcm5	7.894818	*4-Sep*	-8.89482	*Rora*	-2.73697
F2rl2	7.787903	*Rcsd1*	-8.89482	*Fam35a*	-2.73697
Ccnb2	7.787903	*Tmem126a*	-8.84235	*RGD1309676*	-2.68952
Tuft1	7.672425	*A2m*	-8.84235	*Serinc2*	-2.68237
Clcnka	7.672425	*Bves*	-8.84235	*Man1a2*	-2.67807
Zbtb25	7.672425	*Fam19a5*	-8.7879	*Bnip3*	-2.65295
C4bpa	7.672425	*Hspa12b*	-8.7879	*Yy1*	-2.65208
LOC100360880	7.672425	*RGD1311186*	-8.73132	*Spry4*	-2.65208
Col14a1	6.311922	*RGD1565947*	-8.73132	*Mmd*	-2.64989
Leprel2	6.262924	*Mst1*	-8.67243	*Hand2*	-2.64855
Adamtsl3	6.17424	*Amigo2*	-8.67243	*C2cd2l*	-2.64386
Expi	6.050751	*Atf3*	-8.61102	*Exoc5*	-2.63894
Meox2	5.443919	*Chrm2*	-8.61102	*Tspan2*	-2.63819
Tti2	5.393327	*Crabp1*	-8.61102	*Pla1a*	-2.62749
Osr1	5.38184	*LOC682105*	-8.54689	*RT1-CE1*	-2.62449
Serpina3n	4.979057	*Fgf10*	-8.54689	*Ndrg2*	-2.61914
Asb12	4.875433	*Art3*	-8.54689	*Lztfl1*	-2.61891
Clu	4.831837	*Angptl2*	-8.47978	*Ptp4a3*	-2.61891
Rhpn2	4.782902	*Trim29*	-8.47978	*Strbp*	-2.61644
Fibin	4.615576	*Ppm1k*	-8.47978	*Lpl*	-2.60622
Calml4	4.491853	*Nat8l*	-8.47978	*Idi1*	-2.60184
Mycn	4.426265	*Camp*	-8.47978	*Glipr1*	-2.59661
Mtss1	4.403774	*Ednrb*	-8.47978	*Adam19*	-2.57831
Scara5	4.396417	*Mboat2*	-8.40939	*Brp44*	-2.56487
Cdt1	4.357552	*Ebf3*	-8.40939	*Arhgap18*	-2.5486
Efna1	4.247928	*Map4k1*	-8.40939	*Mettl10*	-2.54553
Serpine2	4.241212	*Mpzl2*	-8.33539	*Dapk1*	-2.54432
Gpr88	4.203991	*Cxcl2*	-8.33381	*Gal3st4*	-2.53752
Sulf2	4.201614	*Rbm46*	-8.25739	*Tnfsf9*	-2.52356
Cth	4.197939	*Slc29a4*	-8.25739	*Cenpj*	-2.52356
Akr1c13	4.129889	*Myl1*	-8.25739	*Gadd45g*	-2.51604
Fbn1	4.075849	*Tc2n*	-8.25739	*Asb3*	-2.50416
Flvcr2	4	*Avp*	-8.17493	*Trim16*	-2.50416
Slc7a2	3.908022	*Cpne4*	-8.17493	*Rtn2*	-2.50321
Tst	3.893901	*MGC95152*	-8.17493	*RGD1308019*	-2.5025
Ptpn14	3.859443	*Itgb1bp2*	-8.17493	*Ccl5*	-2.48694
Bmp4	3.840426	*Rbm24*	-8.08746	*Ctxn1*	-2.48618
Dmrt2	3.839804	*Bfsp1*	-8.08746	*Adcy5*	-2.48239
Ubac2	3.829014	*Msi1*	-8.08746	*Ankrd1*	-2.47913
Akr1cl	3.662965	*Sh2d2a*	-8.08746	*Sgk1*	-2.47568
Anxa8	3.615864	*Tasp1*	-8.08746	*Plekho1*	-2.472
Car9	3.61177	*Sncg*	-8.08746	*Exoc8*	-2.45943
Eln	3.584963	*Nppb*	-8.08746	*Srp9*	-2.45672
Srgn	3.571181	*Tnnt1*	-8.08746	*Hbs1l*	-2.4548

Cacna1g	3.537515	*Egfl6*	-8.08746	*Me1*	-2.45404
Cpxm2	3.507031	*Mpp5*	-8.08746	*Entpd5*	-2.44887
Alg3	3.486941	*Acta1*	-7.99435	*Heca*	-2.44057
Egr2	3.459432	*Col4a3*	-7.99435	*Acsl3*	-2.43453
Kbtbd10	3.459432	*Adcy4*	-7.99435	*Traf4*	-2.43296
Plscr2	3.432173	*Wnt5a*	-7.99435	*Grm2*	-2.43296
Cobl	3.407821	*Ckmt2*	-7.99435	*RGD1563888*	-2.43296
Hoxa9	3.392317	*Tnni3*	-7.99435	*Slc3a1*	-2.43296
Fbln1	3.392317	*Slc30a4*	-7.89482	*Cdh11*	-2.43229
Cox6a2	3.374203	*Tspan11*	-7.89482	*Spats2l*	-2.42465
Fmod	3.373272	*Dcaf12l1*	-7.89482	*Cyp7b1*	-2.4196
Rassf4	3.33571	*Rgs7*	-7.89482	*Ehbp1l1*	-2.41801
Il1f10	3.326737	*Rims2*	-7.7879	*Il1r2*	-2.41636
Tk1	3.322635	*Hoga1*	-7.7879	*Gbp5*	-2.41504
LOC303448	3.321928	*Rhbdl3*	-7.7879	*Depdc5*	-2.4021
Gas7	3.296858	*Car6*	-7.7879	*Tbc1d25*	-2.39232
Solh	3.273018	*Pitx2*	-7.7879	*Tpm1*	-2.38774
Atp10a	3.262306	*Actn2*	-7.7879	*Arhgap28*	-2.37851
Kif20a	3.249044	*Stag1*	-7.67243	*Cd53*	-2.37851
Fndc1	3.247928	*Akr1c3*	-7.67243	*RGD1564927*	-2.36923
Pcsk1n	3.233563	*RGD1563349*	-7.67243	*Igfbp3*	-2.36784
Rftn1	3.221848	*Sec31a*	-7.67243	*Sft2d1*	-2.35755
Ccna2	3.169925	*Actg2*	-7.67243	*Ndrg1*	-2.35755
Fbxo5	3.169925	*Slc5a7*	-7.67243	*Lphn1*	-2.35646
Fzd6	3.130495	*Notch4*	-7.67243	*Tnfaip6*	-2.3559
Atf6	3.129283	*Cxcl14*	-7.67243	*Taf1*	-2.35364
LOC691670	3.129283	*Odf1*	-7.67243	*Serpinb9*	-2.35151
Klf5	3.129283	*Cd209d*	-7.67243	*Colec12*	-2.34647
Aox1	3.060025	*Mmp12*	-7.67243	*Serinc5*	-2.33792
Ube2c	3.058894	*Reep1*	-7.67243	*Pla2g16*	-2.33165
Grb14	3.055656	*Spink2*	-7.67243	*Cdo1*	-2.32997
Hip1r	3.054133	*Myl9*	-7.46277	*Nlrp3*	-2.32299
Tgfa	3.052164	*Myh11*	-7.38031	*C2*	-2.3229
Selenbp1	3.022316	*Mmp3*	-6.69367	*Trmt13*	-2.32193
Mcm4	3.00101	*Ier3*	-6.64888	*Gng8*	-2.3156
Fam20c	3.000096	*Mylk*	-6.53904	*Cd248*	-2.30793
Steap3	2.970228	*Pcp4*	-6.34977	*Spin1*	-2.30485
Itpr3	2.963346	*Rarres1*	-6.26789	*Herc4*	-2.29703
Sipa1l1	2.934461	*Pdlim3*	-6.20896	*LOC691921*	-2.29624
Tspan18	2.928726	*Ndufb6*	-6.14692	*Tagln*	-2.29534
Ccnb1	2.925999	*Csf3*	-6.12819	*Gpx3*	-2.29229
Lnx1	2.923764	*Ptges*	-6.06701	*Aoc3*	-2.29126
Gpnmb	2.903272	*Serpinb1a*	-6.01425	*Hapln3*	-2.28951
Siglec10	2.888442	*Il11*	-5.8897	*Osgepl1*	-2.28951

Icam1	2.875886	*Rem1*	-5.81454	*Scoc*	-2.28655
Cdk6	2.847362	*Il33*	-5.748	*Ahi1*	-2.2854
Gjb2	2.829014	*Cnn1*	-5.67827	*Rbm18*	-2.27886
LOC360228	2.813216	*Csrp2*	-5.59582	*Brd2*	-2.27634
Ttc7	2.809776	*Lcp1*	-5.58614	*Ahsp*	-2.26303
Chaf1b	2.807355	*Spink8*	-5.54007	*Tspan12*	-2.25444
Timp3	2.807355	*S100a5*	-5.24882	*Cd59*	-2.24601
Hlx	2.792951	*Tpm2*	-5.14562	*Sclt1*	-2.23704
Slc29a1	2.762366	*Aldh1a2*	-5.11629	*Enpp2*	-2.23386
Dmrta2	2.722466	*Cxcl5*	-5.10934	*Dgcr6*	-2.23282
Asf1b	2.70044	*Dcn*	-5.03872	*Sumf2*	-2.21946
Uap1l1	2.680653	*Galntl4*	-4.97863	*Mettl9*	-2.21719
Ptgis	2.667349	*Vwa1*	-4.91514	*Plk4*	-2.21466
Nmnat3	2.664453	*Il1b*	-4.88336	*Crem*	-2.21266
Cd200	2.656479	*Il17b*	-4.80887	*Ube2m*	-2.20766
Psat1	2.655576	*Slc16a3*	-4.70411	*Bbx*	-2.19265
Trim24	2.651005	*Ebf1*	-4.68732	*Il13ra2*	-2.19265
Pkmyt1	2.643856	*Mgst3*	-4.62128	*Hmgn5*	-2.18612
Tjp2	2.624544	*Ccl20*	-4.5102	*Ndn*	-2.1763
C1s	2.620988	*Spry1*	-4.49813	*Mfap4*	-2.17463
Slc1a3	2.619568	*C1qtnf7*	-4.49248	*LOC683788*	-2.16074
Trim37	2.584963	*Hspb7*	-4.46347	*Nudcd2*	-2.15094
Zbtb7c	2.584963	*Dhrs3*	-4.35859	*LOC682999*	-2.14708
Mcm2	2.545129	*Fgf2*	-4.32193	*Scly*	-2.14684
Ubc	2.543515	*Pla2g7*	-4.27935	*Ccdc72*	-2.13644
Abcb1a	2.537028	*Tmem88*	-4.24793	*Cald1*	-2.13531
Sgcg	2.528958	*Bmp2*	-4.22391	*Bmper*	-2.13024
Me3	2.523562	*Tubb3*	-4.1907	*Zfp386*	-2.1273
RGD1565469	2.523562	*Pla2g4a*	-4.16579	*Imp3*	-2.12314
Lcn2	2.504944	*Lmod1*	-4.16012	*Jkamp*	-2.11548
MGC105649	2.501055	*Plk3*	-4.12199	*Larp1b*	-2.10812
Haus4	2.475627	*Ptk7*	-4.09201	*Lrrfip2*	-2.0984
Glis2	2.473252	*Bace2*	-4.08746	*Lamc2*	-2.09796
Agtr1a	2.465982	*Itga8*	-4.08121	*Tmem123*	-2.09727
C3	2.438444	*Nexn*	-4.0493	*Prss35*	-2.09443
Sema5a	2.424649	*Prl8a9*	-4.04439	*RGD1563982*	-2.08246
Ces5a	2.42393	*Senp8*	-4.04439	*Chst10*	-2.07506
Ptpn21	2.4087	*Micalcl*	-4	*Figf*	-2.07039
Hp	2.407871	*Ckb*	-3.99382	*Rnf168*	-2.06413
Enpp3	2.406793	*Cotl1*	-3.98411	*Ift20*	-2.05526
Zfp608	2.402098	*Mipep*	-3.9542	*Tes*	-2.05398
Nov	2.40012	*Tp53i11*	-3.91663	*Sipa1l2*	-2.05209
RGD1309079	2.385645	*Tbc1d9*	-3.90689	*Uaca*	-2.05136
Tap2	2.377914	*Pde2a*	-3.8929	*Gfpt2*	-2.04543

Sgsm2	2.370146	*Vstm4*	-3.87447	*Taf13*	-2.04525
Spp1	2.369376	*Tgm2*	-3.86011	*Creg1*	-2.04439
Lmo2	2.36257	*Ctsc*	-3.82901	*Crlf1*	-2.04232
Tgm1	2.353637	*Mex3b*	-3.80735	*Bscl2*	-2.03916
Birc5	2.35286	*Palmd*	-3.77362	*Itpr1*	-2.03562
Rassf9	2.35216	*Lum*	-3.75789	*Klc4*	-2.03126
Grn	2.347461	*Ctsz*	-3.73767	*15-Sep*	-2.03086
Phgdh	2.334451	*Mcat*	-3.70207	*Cklf*	-2.03061
Wnk4	2.323342	*Etv1*	-3.70174	*B3galnt2*	-2.02908
Cdkn2c	2.32314	*Shd*	-3.64386	*Sh3bgrl*	-2.02745
Cdc20	2.321928	*Cacna2d1*	-3.62449	*Tifa*	-2.02702
Eepd1	2.321928	*Duox1*	-3.6199	*Rbm7*	-2.02367
Ints7	2.321928	*Postn*	-3.60643	*Cdr2*	-2.02283
Oas1f	2.321928	*Acta2*	-3.60269	*Kif5b*	-2.02147
Matn4	2.321222	*Ptgs2*	-3.58496	*B4galt3*	-2.02106
Prps2	2.301784	*Lrrc15*	-3.58496	*Acat1*	-2.01851
Tgfbrap1	2.300886	*Hspb6*	-3.55835	*Rp2*	-2.01651
RGD1309437	2.293647	*Iscu*	-3.53179	*Pdcd5*	-2.01311
Slc17a9	2.28649	*Ppp1r3c*	-3.52418	*Dynlt3*	-2.01224
Flot1	2.271018	*Abcg1*	-3.51245	*Ephx1*	-2.00434
Nfib	2.264801	*Rpl11*	-3.48867	*Pawr*	-2.00343
St6galnac2	2.258142	*Isg15*	-3.46803	*Nt5dc1*	-2.00163
Ext2	2.255147	*Plcb4*	-3.46643	*Hspb1*	-2.00132
Col1a1	2.232254	*Tmem106a*	-3.45943	*Plekha5*	-2.00118
Spc25	2.222392	*Vash2*	-3.45943	*Lipogenin*	-2.00106
Mfsd10	2.20847	*G0s2*	-3.43024	*Bola1*	-2.00088
Plekha4	2.205159	*Ms4a6b*	-3.39333	*Gstk1*	-2.00066
Fos	2.201634	*Pcca*	-3.39232	*Col7a1*	-2
Ube2l3	2.194843	*Acp5*	-3.39232	*Crcp*	-2
Foxo4	2.19397	*Gucy1b3*	-3.37606		
Ucp2	2.183874	*Ina*	-3.36923		
Stom	2.18195	*Kcnk12*	-3.36923		
Ccdc80	2.179991	*Cxcl13*	-3.35216		
Plscr1	2.171103	*Nos2*	-3.34407		
Pla2g2e	2.169925	*Fam101b*	-3.32293		
Hmox2-ps1	2.161581	*Dnajc7*	-3.32193		
Dusp10	2.157009	*Gstm7*	-3.32193		
Six1	2.150942	*Fez1*	-3.32193		
Gm2a	2.145512	*Nid2*	-3.31137		
Zfp521	2.129283	*Sfrp4*	-3.30371		
Pnkp	2.100949	*Fzd8*	-3.29912		
LOC100366030	2.100243	*Sh3kbp1*	-3.2854		
Il1rn	2.076764	*Osr2*	-3.27448		
Rrm2	2.071399	*Ms4a7*	-3.27302		

Slit3	2.047257	*Cgnl1*	-3.24942
Ror2	2.045957	*Acsl6*	-3.24793
Nfatc1	2.045679	*Pdpn*	-3.22683
Mcm6	2.035245	*Cox4i2*	-3.19834
Mri1	2.030536	*Tfpi2*	-3.182
Gpc4	2.025253	*Ccl3*	-3.16993
Slc7a1	2.023757	*Mb*	-3.16993
Tnfrsf9	2.019961	*Nhs*	-3.16993
Man2b2	2.013655	*Rarres2*	-3.15423
Comt	2.012375	*Hdac11*	-3.12883
Eif4ebp2	2.000922	*Esm1*	-3.12736
Impa2	2	*Phf14*	-3.12477
Neu1	2	*MGC116197*	-3.0976
